# Effectiveness of pharmacotherapy for smoking cessation: protocol for umbrella review and quality assessment of systematic reviews

**DOI:** 10.1186/s13643-018-0878-3

**Published:** 2018-11-24

**Authors:** Alemu S. Melka, Catherine L. Chojenta, Elizabeth G. Holliday, Deborah J. Loxton

**Affiliations:** 1 Department of Public Health, Institute of Health Sciences, Wollega University, Nekemte, Ethiopia; 2 Research Centre for Generational Health and Ageing, Faculty of Health and Medicine, The University of Newcastle, Newcastle, Australia; 3 School of Medicine and Public Health, Faculty of Health and Medicine, The University of Newcastle, Newcastle, Australia

**Keywords:** Smoking cessation, Pharmacotherapy, Nicotine replacement therapy

## Abstract

**Background:**

In the long term, smoking cessation can decrease the risk of cancer, stroke, and heart attacks and improve overall survival. The aim of the proposed umbrella review is to summarize existing systematic reviews that assessed the effects of pharmacological interventions for smoking cessation and to evaluate the methodological quality of previously conducted systematic reviews.

**Methods:**

Databases such as the Cochrane Library, PubMed, MEDLINE, EMBASE, CINAHIL PsychINFO Web of Science, Conference Papers Index, Scopus, and Google Scholar will be used to retrieve reviews. Systematic reviews which included only randomized control trials will be considered in this review. The primary outcome will be prolonged abstinence from smoking for a minimum of 6 months follow-up, and the secondary outcome will be point abstinence rate from smoking of less than 6 months follow-up but more than 7 days. Methodological quality of the included reviews will be assessed using the Assessment of Multiple Systematic Reviews 2 (AMSTAR 2) tool, which contains 16 domains. Two authors will screen the titles and abstracts of all reviews obtained by the search strategy, assess the full text of selected articles for inclusion, and extract data independently. The quality appraisal will be also assessed by two authors (AM, CC) independently, and Cohen’s Kappa statistic will be used to assess inter-ratter agreement. The findings of the study will be narrated qualitatively to describe the effect of different pharmacotherapy on smoking cessation.

**Discussion:**

The World Health Organization recommends treatment of tobacco dependence as one approach in its comprehensive tobacco control policy. To date, many trials and systematic reviews have been conducted to assess the effectiveness of pharmacotherapy for smoking cessation. Therefore, the findings of the umbrella review will improve clinical decision-making and be used as a baseline for future studies.

**Systematic review registration:**

PROSPERO CRD42017080906

**Electronic supplementary material:**

The online version of this article (10.1186/s13643-018-0878-3) contains supplementary material, which is available to authorized users.

## Background

Globally, in 2012, the prevalence of daily tobacco smoking among men and women aged 15 and over was 31.1% and 6.2%, respectively [1]. Smoking seriously affects almost all organs in the body. Tobacco smoking can lead to many short- and long-term health effects including lung and other organ cancers, chronic bronchitis, emphysema, stroke, and heart attack [2]. Tobacco smoking is responsible for 90% of all cases of lung cancer and 90% of all deaths due to chronic obstructive pulmonary disease (COPD) [3]. According to the World Health Organization, tobacco smoking kills about six million people globally per annum [4]. Second-hand smoke contains hundreds of chemicals responsible for diseases such as respiratory disorders, cancer, and cardiovascular disease. Combustible chemicals found in tobacco smoke are responsible for disorders such as cancer, cardiovascular, and pulmonary diseases, through mechanisms that involve DNA damage, inflammation, and oxidative stress [5]. Globally, second-hand smoking affects women and children more than men [6, 7]. Tobacco-related disability-adjusted life years (DALYs) account for 4% of the global burden with the burden significantly higher for developed nations [8].

Tobacco contains about 4000 chemicals; nicotine is one of the chemicals contained in tobacco, and it is responsible for addictive behavior [9]. During smoking, the nicotine components are absorbed through the mucous membrane and enter the brain through the bloodstream. Upon entering the brain, nicotine stimulates the release of epinephrine and dopamine which in turn increases blood pressure, heartbeat, and respiration rate and produces pleasurable feelings [3, 9].

In the long term, smoking cessation can decrease the risk of cancer, stroke, and heart attacks and can also improve survival [3, 10]. Smoking cessation can also decrease the risk of respiratory infections such as pneumonia, influenza, and chronic obstructive pulmonary disease. [11]. Kahler et al. and Eddy et al. have shown that quitting smoking is associated with significant reductions in risk of COPD, myocardial infarctions, stroke, and coronary heart disease [12, 13]. Moreover, smoking cessation can improve health-related quality of life (physical, psychological, and social functioning in relation to health) [14].

A range of smoking cessation interventions is available which can be broadly categorized as motivational, behavioral/psychological, and pharmacological. The World Health Organization recommends that countries prioritize different smoking cessation strategies depending on their available resources, national health system, and political will to implement the cessation strategies [15]. Treatment of tobacco smoking, like any other forms of substance dependence, necessitates pharmacological interventions to minimize cravings and the treatment of withdrawal symptoms associated with nicotine dependence [9]. Nicotine replacement therapies (NRT) in different formulations such as inhalation, patches, gums, nasal sprays, and lozenges can be used for treatment of withdrawal symptoms after smoking cessation. Since the nicotine concentration in NRT is low compared to tobacco, it has a low addiction rate [3].

Amfebutamone (bupropion) represents the first non-nicotine drug used for the treatment of nicotine dependence. Amfebutamone works by antagonizing nicotine receptors and inhibits the reuptake of epinephrine, dopamine, and serotonin, thus reducing withdrawal symptoms [16–18]. Varenicline is a nicotine receptor partial agonist that blocks nicotine receptors by binding into α_4_β_2_ nicotinic acetylcholine receptors and moderately releases dopamine, thus reducing the craving and withdrawal symptoms associated with an absence of nicotine [19].

The success of smoking cessation was associated with factors such as male gender, smoking frequency, alcohol dependence, a household ban on smoking, living with a smoker, and having close friends who smoke [20, 21]. The most common adverse effects of NRT include insomnia, headache, vomiting, dizziness, palpitation, anxiety, and depression. Bupropion and varenicline are also associated with side effects like insomnia, dry mouth, agitation, and nausea [22, 23]. A systematic review identified a positive relationship between weight gain and smoking cessation [24]. Researchers have recommended the importance of weight management interventions along with smoking cessation interventions [25]. There are enzymes produced as a result of cigarette smoking responsible for the metabolism of drugs like clozapine. Therefore, smoking cessation can lead to a toxic side effect as a result of an increase in drug plasma concentration and requires monitoring and reduction in the dose of drugs [26].

Although most of the previous trials and systematic reviews confirmed the effectiveness of behavioral interventions [27, 28] for smoking cessation, the findings are not consistent for pharmacological interventions. Therefore, the proposed umbrella review will provide a summary of the evidence on the effectiveness of different types of pharmacotherapy. To date, many trials and systematic reviews have been conducted to assess the effectiveness of pharmacotherapy for smoking cessation. The proposed umbrella review of existing systematic reviews will present evidence from previous systematic reviews to help inform decision-makers and clinicians.

### Objectives

The proposed umbrella review will synthesize findings of previous reviews in order to evaluate the effects of different pharmacotherapies for smoking cessation and assess consistency of conclusions among previous systematic reviews. The proposed umbrella review will summarize the effects of pharmacological interventions reported by each review of smoking cessation, specifically addressing the following objectives:

- To summarizing existing systematic reviews that assessed the effects of pharmacological interventions for smoking cessation
- To assess the methodological quality of previously conducted systematic reviews

## Methods

### Protocol registration and reporting of findings

The protocol of this review followed the guidelines of Preferred Reporting Items for Systematic Review and Meta-analysis Protocols (PRISMA-P) [29]. The PRISMA-P checklist is available as Additional file 1. The protocol was registered in PROSPERO with registration numberCRD42017080906:http://www.crd.york.ac.uk/PROSPERO/display_record.php?ID=CRD42017080906. The findings of the review will be reported in accordance with the recommendation of Preferred Reporting Items for Systematic Review and Meta-analysis (PRISMA) [30]. If we intend to modify this protocol, we will give the date of each amendment, describe the change, and give the reason for the change.

### Inclusion and exclusion criteria

Since the primary aim of the proposed umbrella review is to identify the effects of pharmacological interventions on smoking cessation, only reviews that included randomized control trials will be reviewed. Since tobacco cessation interventions are mostly targeted at adults aged 15 and over, in the proposed umbrella review, we will include studies of young people and adults aged 15 and over who were smokers. In the proposed umbrella review, we will consider only systematic reviews/systematic reviews with network meta-analysis that include primary studies with randomized control trial designed to assess the effect of pharmacotherapy for smoking cessation. Systematic reviews published 10 years before the last date of review will be included in the proposed review. The umbrella review will include only reviews for which full text is available. The primary outcome will be prolonged abstinence from smoking for a minimum of 6 months follow-up, and the secondary outcome will be point abstinence rate from smoking of less than 6 months follow-up but more than 7 days. We will prefer biochemical methods over self-reported verification. The control or comparison groups used will be either placebo, behavioral interventions, or pharmacotherapy. The review will include only those reviews which report pooled effects of the included studies through meta-analysis or network meta-analysis.

The umbrella review will include only studies published in English. If a review is an update of a previous review, the latest updates will be considered and the oldest versions will be excluded. Reviews which assessed combined pharmacotherapy and behavioral interventions will be excluded unless the reviews report the effect of pharmacotherapy separately, in which case, these reviews will be included. The summary of inclusion criteria based on population, intervention, comparator, outcome, and study design (PICOS) is presented in Table 1.

**Table 1:** Population, intervention, comparator, outcome, and study design (PICOS) elements

PICOS elements	Inclusion and exclusion criteria
Population	Young people and adults aged 15 and over who were smokers
Intervention	Reviews assessed only the effect of pharmacotherapy on smoking cessation (nicotine, bupropion and varenicline and combined therapy). Reviews which assessed combined pharmacotherapy and behavioral interventions will be excluded.
Comparator	The control may be either standard care or placebo, behavioral intervention or no intervention
Outcome	Reviews that assessed abstinence from smoking for a minimum of 6 months follow-up for prolonged abstinence rate and less than 6 months but greater than 7 days for point prevalence rate.
Study design	Reviews that include only randomized control trials published in English. Reviews that include observational studies will be not included.

### Information source and search strategy

To trace related reviews, databases such as the Cochrane Library, PubMed, MEDLINE, EMBASE, CINAHIL, and PsychINFO will be searched. Moreover, Web of Science, Conference Papers Index, Scopus, and Google Scholar will be used. Additional reviews will be sought using the reference lists of the retrieved articles. Additional articles will be traced from daily email alerts from MEDLINE database throughout the review process. The search strategy will be developed in consultation with a senior librarian. Different keywords/search terms will be used to access reviews from the database including “smoking cessation,” “smoking abstinence,” “Pharmacotherapy,” “Nicotine replacement therapy (NRT),” “bupropion,” “Varenicline,” “combination therapy,” “non-nicotine drug,” “nicotine receptor partial agonist,” “meta-analysis,” and “Systematic review.” The search strategy for MEDLINE is found in Additional file 2.

### Data collection processes

First, irrelevant studies not fulfilling the inclusion criteria for the systematic review will be excluded by reading the title and then by reading the abstract of the articles. Next, the full articles will be accessed and those articles which do not fit the objective of the review will be omitted. The excluded studies will be recorded along with the reason for exclusion at each stage. If additional information is required, the primary author of the published paper will be contacted. Cochrane data abstraction format will be used to extract information from the studies. Two authors (AM, CC) will carry out the following processes independently: screen the titles and abstracts of all publications obtained by the search strategy and assess the full text of selected articles for against inclusion criteria. Any discrepancy that arises between the two authors will be resolved through discussion. In case the authors not able to reach agreement, a third author will be consulted for assistance (LH). Cochrane data abstraction form will be used to extract data on the objectives of the study, study design, study inclusion and exclusion criteria, the number of articles and participants included, participant characteristics, intervention, control, outcome and pooled effects among others. The data extraction form is found in Additional file 3.

### Assessment of methodological quality

Methodological quality of the included reviews will be assessed using the Assessment of Multiple Systematic Reviews 2 (an update of AMSTAR) tool, which contains 16 domains [31]. The tool includes 10 items from the original AMSTAR tool. Two items were created by splitting a single item from the original AMSTAR tool [32]. In total, four domains were added in AMSTAR 2 which were not found in the original tool. The response option for most domains consists of “yes” and “no” while some domains contain the third option “partial yes.” AMSTAR has been shown to have good inter-rater reliability to assess the quality of systematic reviews. From the 16 AMSTAR tool items, 7 were critical domains upon which the quality rating of individual systematic reviews depends (Table 2). Based on the overall score, the quality of each systematic review will be rated as high, moderate, low, and critically low. Table 2 presents the criteria to rate the quality of systematic review. The AMSTAR 2 checklist is found in Additional file 4. Scores for each item will be reported separately for each systematic review. The quality assessment of the included systematic review will be conducted by two independent reviewers (AM, CC), and any disagreement between the two reviewers will be resolved with discussion. In case the authors not able to reach agreement, a third author will be consulted for assistance (LH). Cohen’s Kappa statistic will be used to assess inter-rater agreement. A Kappa value below 60% indicates inadequate agreement among the raters [33]. Studies will not be excluded based on their quality, but the assessment serves to judge the strength of evidence generated by the included studies.

**Table 2:** Quality rating criteria

Quality rating	Criteria	AMSTAR 2 critical domains
High	No or one non-critical weakness	• Protocol registered before commencement of the review (item 2) • Adequacy of the literature search (item 4) • Justification for excluding individual studies (item 7) • Risk of bias from individual studies being included in the review (item 9) • Appropriateness of meta-analytical methods (item 11) • Consideration of risk of bias when interpreting the results of the review (item 13) • Assessment of presence and likely impact of publication bias (item 15)
Moderate	More than one non-critical weakness	
Low	One critical flaws with or without non-critical weakness	
Critically low	More than one critical flaw with or without non-critical weakness	

### Data synthesis

In this review, a meta-analysis will not be conducted because data from individual studies are likely to be represented more than once across the systematic reviews, and this will likely lead to over or underestimations of the true effect size [34]. The required information will be collected using a pretested checklist adopted from the Cochrane data abstraction format, according to the objective of the review [35]. Narrative synthesis method will be employed to show the effects of different pharmacotherapies on smoking cessation. The Narrative presentation will include the overall effect size reported by systematic review authors along with statistical heterogeneity and methodological quality. Evidence will be summarized in a table which will present the types of intervention, comparators, outcome measures, number of participants, number of included primary studies, and pooled results from each review, heterogeneity, and the review author’s conclusions. To calculate the degree of overlap, we will calculate corrected covered area (CCA) by dividing the frequency of repeated occurrence of index publication in other reviews by the product of index publications and reviews less the number of index publications. The CCA will be rated as follows: CCA less than 5 will be rated as slight overlap, 6–10 moderate overlap, 11–15 high overlap, and greater than 15 as a very high overlap [36]. The degree of overlap will be stated as a limitation while interpreting the findings.

### Assessing confidence in evidence

The quality of evidence reported by the systematic review authors will be reported for primary studies. If the systematic reviews fail to report the quality of evidence, we will assess the risk of bias using Grading of Recommendations Assessment, Development and Evaluation (GRADE) measures.

## Discussion

Systematic reviews are considered as an important source of information for clinicians and policy makers because combining the findings from a series of studies can provide a more accurate and reliable evidence base through improved statistical power. Moreover, systematic reviews are an important tool for the development of guidelines and strategies for medical practice and suggesting directions for new research [37, 38]. Clinicians and decision-makers need to assure themselves that the basic approaches and methods used to collect and combine the findings of individual studies are relevant and sound before using the evidence for patient care and policy development [38, 39]. Review of systematic reviews help as a method to providing a higher level combination of evidence to wide public health problems [40].

The World Health Organization recommends including treatment of tobacco dependence as one strategy of its comprehensive tobacco-control policy, along with measures such as taxation and price policies, advertising restrictions, dissemination of information, and establishment of smoke-free public places [15]. Therefore, in the proposed review of reviews, we will be summarizing existing systematic reviews that assessed the effects of pharmacological interventions for smoking cessation and also conduct methodological quality of the included reviews. The findings of our review will improve clinical decision-making and will be used as a baseline for future studies.

## Additional files

Additional file 1: PRISMA-P checklist (DOCX 66 kb)

Additional file 2: Search strategy in Medline (DOCX 19 kb)

Additional file 3: Data abstraction form (DOCX 23 kb)

Additional file 4: Quality assessment tool (AMSTRA 2) (PDF 173 kb)

## Abbreviations

AMSTAR: Assessment of Multiple Systematic Reviews
COPD: Chronic obstructive pulmonary disease
NRT: Nicotine replacement therapy
PRISMA: Preferred Reporting Items for Systematic Review and Meta-analysis
PROSPERO: International prospective register of systematic reviews
R-AMSTAR: Revised Assessment of Multiple Systematic Reviews
