# Supplementary material for: Effectiveness of pharmacotherapy for smoking cessation: protocol for umbrella review and quality assessment of systematic reviews
Source: Syst Rev. 2018 Nov 24;7:210. doi: 10.1186/s13643-018-0878-3 (PMC6260841; doi:10.1186/s13643-018-0878-3)
Supplement: Supplementary file 2 — Search strategy in Medline (DOCX 19 kb) [file 13643_2018_878_MOESM2_ESM.docx]

**Additional file 2**: Search Strategy in Medline

1. (Intervention* or therapy* or treat*).ti,ab.
2. Pharmacotherapy.ti, ab.
3. Nicotine replacement therapy .ti,ab.
4. (Nicotine gums or nicotine patch or nicotine nasal spray or nicotine inhaler or nicotine lozenge).ti,ab.
5. Bupropion .ti,ab.
6. Varenicline .ti,ab.
7. Combination therapy .ti,ab.
8. "Tobacco Use Cessation Products"/
9. (Non-nicotine drug or nicotine receptor partial agonist).ti,ab.
10. 1 or 2 or 3 or 4 or 5 or 6 or 7 or 8 or 9
11. ((quit* or stop* or abstin* or abstain* or reduc* or ceas* or cessation) adj3 (smoke* or tobacco*)).ti,ab.
12. "tobacco use cessation"/ or smoking cessation/
13. 11 or 12
14. meta-analysis/
15. Meta-Analysis as Topic/
16. (Metaanalys* or meta analys*).ti,ab.
17. (Systematic adj2 (review* or overview*)).mp.
18. 14 or 15 or 16 or 17
19. 10 and 13 and 18
20. limit 19 to (abstracts and English language and full text and humans)
