# Supplementary material for: Effectiveness of pharmacotherapy for smoking cessation: protocol for umbrella review and quality assessment of systematic reviews
Source: Syst Rev. 2018 Nov 24;7:210. doi: 10.1186/s13643-018-0878-3 (PMC6260841; doi:10.1186/s13643-018-0878-3)
Supplement: Supplementary file 3 — Data abstraction form (DOCX 23 kb) [file 13643_2018_878_MOESM3_ESM.docx]

**Additional file 3**: Data extraction form

| Items | Extracted data |
| --- | --- |
| Reviewer |  |
| Identification features of the study: |  |
| Author |  |
| review title |  |
| Year range considered for inclusion |  |
| Type of publication (e.g. journal article, conference abstract) |  |
| Year of publication |  |
| Journal name: |  |
| Last search date |  |
| Region/state/country |  |
| Source of funding |  |
| Aim/objectives of the study |  |
| Study design included |  |
| Other inclusion criteria |  |
| Study exclusion criteria |  |
| Protocol registered |  |
| Database searched |  |
| Number of articles included |  |
| Number of articles included in meta-analysis |  |
| Participant characteristics (age, sex, number of participants) |  |
| Quality assessment tool and source |  |
| Cochrane Collaboration reviews Vs not |  |
| Type of intervention included |  |
| Control |  |
| Intervention (focus, duration, intensity, delivery format) |  |
| Duration of intervention before measuring outcome |  |
| Outcome variable |  |
| Definition of smoking prevention or cessation |  |
| `Validation of cessation (biological markers or self-report). |  |
| Total Sample size |  |
| Type of analysis used in review |  |
| Pooled effect |  |
| Findings |  |
| Adverse Events |  |
| Sub-group analysis/sensitivity test criteria and findings |  |
| Overall quality of included studies |  |
| Quality of the evidence |  |
| Reported heterogeneity of the studies |  |
| Reporting guideline used (ex. PRISMA) |  |
| Key conclusions of study authors: Further study information requested |  |
| Reviewers comments on methodology, limitations, generalisability that you have after reading the paper |  |
| Reported publication bias |  |
